# The median effective concentration of epidural ropivacaine with different doses of dexmedetomidine for motor blockade: an up-down sequential allocation study

**DOI:** 10.3389/fmed.2024.1413191

**Published:** 2024-08-05

**Authors:** Ji-Xiang Wan, Chao Lin, Zhi-Qiang Wu, Duan Feng, Yuan Wang, Fang-Jun Wang

**Affiliations:** Department of Anesthesiology, Affiliated Hospital of North Sichuan Medical College, Nanchong, China

**Keywords:** dexmedetomidine, epidural, median effective concentration, motor blockade, ropivacaine

## Abstract

**Study objective:**

Recent studies have shown that dexmedetomidine can be safely used in peripheral nerve blocks and spinal anesthesia. Epidural administration of dexmedetomidine produces analgesia and sedation, prolongs motor and sensory block time, extends postoperative analgesia, and reduces the need for rescue analgesia. This investigation seeks to identify the median effective concentration (EC_50_) of ropivacaine for epidural motor blockade, and assess how incorporating varying doses of dexmedetomidine impacts this EC_50_ value.

**Design:**

Prospective, double-blind, up-down sequential allocation study.

**Setting:**

Operating room, post-anesthesia care unit, and general ward.

**Interventions:**

One hundred and fifty patients were allocated into five groups in a randomized, double-blinded manner as follows: NR (normal saline combined with ropivacaine) group, RD_0.25_ (0.25 μg/kg dexmedetomidine combined with ropivacaine) group, RD_0.5_ (0.5 μg/kg dexmedetomidine combined with ropivacaine) group, RD_0.75_ (0.75 μg/kg dexmedetomidine combined with ropivacaine) group, RD_1.0_ (1.0 μg/kg dexmedetomidine combined with ropivacaine) group. The concentration of epidural ropivacaine for the first patient in each group was 0.5%. Following administration, the patients were immediately placed in a supine position for observation, and the lower limb motor block was assessed every 5 min using the modified Bromage score within 30 min after drug administration. According to the sequential method, the concentration of ropivacaine in the next patient was adjusted according to the reaction of the previous patient: effective motor block was defined as the modified Bromage score > 0 within 30 min after epidural administration. If the modified Bromage score of the previous patient was >0 within 30 min after drug administration, the concentration of ropivacaine in the next patient was decreased by 1 gradient. Conversely, if the score did not exceed 0, the concentration of ropivacaine in the next patient was increased by 1 gradient. The up-down sequential allocation method and probit regression were used to calculate the EC_50_ of epidural ropivacaine.

**Measurements:**

Adverse events, hemodynamic changes, demographic data and clinical characteristics.

**Main results:**

The EC_50_ of epidural ropivacaine required to achieve motor block was 0.677% (95% *CI*, 0.622–0.743%) in the NR group, 0.624% (95% *CI*, 0.550–0.728%) in the RD_0.25_ group, 0.549% (95% *CI*, 0.456–0.660%) in the RD_0.5_ group, 0.463% (95% *CI*, 0.408–0.527%) in the RD_0.75_ group, and 0.435% (95% *CI*, 0.390–0.447%) in the RD_1.0_ group. The EC_50_ of the NR group and the RD_0.25_ group were significantly higher than that of the RD_0.75_ and the RD_1.0_ groups, and the EC_50_ of the RD_0.5_ group was significantly higher than that of the RD_1.0_ group.

**Conclusion:**

The EC_50_ of epidural ropivacaine required to achieve motor block was 0.677% in the NR group, 0.624% in the RD_0.25_ group, 0.549% in the RD_0.5_ group, 0.463% in the RD_0.75_ group, and 0.435% in the RD_1.0_ group. Dexmedetomidine as an adjuvant for ropivacaine dose-dependently reduce the EC_50_ of epidural ropivacaine for motor block and shorten the onset time of epidural ropivacaine block. The optimal dose of dexmedetomidine combined with ropivacaine for epidural anesthesia was 0.5 μg/kg.

## 1 Background

Epidural anesthesia is extensively applied across various surgical domains, including urology, lower abdominal and lower limb orthopedic procedures (1, 2). Epidural anesthesia is administered to provide intraoperative surgical anesthesia and postoperative analgesia (3). It not only diminishes the incidence of complications by mitigating the perioperative stress response but also facilitates early mobilization through alleviation of postoperative pain, thereby preventing the development of lower limb venous thrombosis (4–7). However, epidural anesthesia frequently results in inadequate or superficial blockade (8). In order to overcome these weaknesses, we usually enhance the effect of epidural anesthesia by adding opioids (9, 10). Opioids like fentanyl and sufentanil, as adjuvants for epidural administration, provide good analgesic effects (11), but can cause adverse effects such as itching, nausea, vomiting, and respiratory depression (12, 13).

Dexmedetomidine is a highly selective α_2_ adrenergic receptor agonist (14). Clinical studies have shown that dexmedetomidine can be safely used for peripheral nerve blockade and spinal anesthesia (15–19). Recent investigations have demonstrated that the administration of dexmedetomidine via epidural route not only induces analgesia and sedation (20) but also intensifies the efficacy of motor and sensory blockades (21, 22). Nonetheless, as the dosage of epidural dexmedetomidine escalates, there is a corresponding increase in the occurrence of adverse effects (23). Consequently, investigating the optimal dosage of dexmedetomidine in combination with ropivacaine for epidural administration is imperative.

This study is designed to identify the EC_50_ of ropivacaine for epidural motor blockade, as well as to assess the impact of various doses of dexmedetomidine on this EC_50_ value. Concurrently, it aims to offer guidance on the optimal dosage of dexmedetomidine when used as an adjunct to ropivacaine in epidural anesthesia.

## 2 Methods

### 2.1 Study design

We conducted a prospective, double-blind, up-down sequential allocation study to determine the EC_50_ of ropivacaine for motor blockade in epidural anesthesia when combined with different does of dexmedetomidine. The study received approval from the Ethics Committee of the Affiliated Hospital of North Sichuan Medical College (2022ER083-1) and was registered at the Chinese Clinical Trial Registry (registration number: ChiCTR2200065955, registration date: November 19, 2022). All participants in the clinical trial provided written informed consent and were treated at the Affiliated Hospital of North Sichuan Medical College. The enrollment of the first patient took place on November 25, 2022.

Study subjects who met the following inclusion criteria were considered: all patients scheduled for elective urological surgery, aged from 18 to 65 years, with body mass index (BMI) more than or equal to 18.5 kg/m^2^ and less than 30 kg/m^2^, American Society of Anesthesiologists (ASA) physical status grade I or II, were enrolled from November 2022 to March 2023. Exclusion criteria included patients with a history of drug addiction, those whose patients or their families refusing to participate, those with anticipated difficulty in regional anesthesia, allergies to the study drug, and those with bradycardia. Also excluded were individuals with severe cardiovascular or pulmonary diseases, liver or kidney dysfunction, spinal deformity, and coagulation disorders. Further exclusions were patients with infection at the site of epidural puncture, cognitive dysfunction, hearing impairment, lower limb motor disorders, or sensory abnormalities.

All patients were divided into five groups using computer-generated random numbers. These random numbers were marked on cards and placed in sealed envelopes within an opaque box. Upon the patient’s arrival in the operating room, the anesthesia nurse randomly drew an envelope and administered the test drug according to the group in the envelope, which used sealed envelopes indicating the allocation: normal saline combined with ropivacaine (Ropivacaine hydrochloride injection, 10 mL:100 mg, AstraZeneca AB, Sweden) (NR) group, 0.25 μg/kg dexmedetomidine combined with ropivacaine (RD_0.25_) group, 0.50 μg/kg dexmedetomidine combined with ropivacaine (RD_0.5_) group, 0.75 μg/kg dexmedetomidine combined with ropivacaine (RD_0.75_) group, 1.0 μg/kg dexmedetomidine combined with ropivacaine (RD_1.0_) group. The anesthesia nurse completed the drug preparation and gave it to the anesthesiologist in this study. After the experiment, the anesthesiologist showed the data back to the statistician. Study participants and the investigators who performed outcome assessments were blinded to the grouping during the study period.

### 2.2 Anesthesia

Prior to surgery, all patients underwent an 8-h fasting period for solid food and a 2-h fasting period for clear liquids. Additionally, they did not receive any preoperative medication. Upon arrival in the operating room, a secure intravenous access was established. Simultaneously, patients were preloaded with 10 mL/kg of lactated Ringer’s solution and continuously monitored with electrocardiogram (ECG), oxygen saturation, and noninvasive blood pressure (NIBP). A face mask was used to administer a supplemental oxygen flow rate of 6 L/min to all patients. The study drug was prepared by an anesthesia nurse and then handed to another anesthesiologist who was unaware of its composition. Epidural anesthesia was performed with patients positioned in the left lateral decubitus position in the L_2_-L_3_ intervertebral space. The epidural space was confirmed using the loss of resistance technique. An epidural catheter was inserted 4–5 cm into the epidural space and securely fixed. To rule out the possibility of intrathecal injection, 3 mL of 2% lidocaine was administered as a test dose after negative aspiration for blood and cerebrospinal fluid, no adrenaline was added to the test dose. The NR group received a 15 mL mixture of normal saline and ropivacaine, whereas the RD_0.25_, RD_0.5_, RD_0.75_ and RD_1.0_ groups were administered a 15 mL combination of dexmedetomidine and ropivacaine, the dosages of dexmedetomidine for these groups were 0.25 μg/kg, 0.5 μg/kg, 0.75 μg/kg, and 1.0 μg/kg, respectively. The mixed solution was then continuously infused into epidural space at a rate of 1 mL/s in a single administration. The concentration of epidural ropivacaine for the first patient in each group was 0.5% (24). Following administration, the patients were immediately placed in a supine position for observation, and the lower limb motor block was assessed every 5 min using the modified Bromage score (Bromage 0 = fully able to flex knees and feet; Bromage 1 = just able to move knees; Bromage 2 = unable to move knees, able to move feet only; Bromage 3 = unable to move knees and feet) (25) within 30 min after drug administration. According to the sequential method (26), the concentration of ropivacaine in the next patient was adjusted according to the reaction of the previous patient: effective motor block was defined as the modified Bromage score > 0 within 30 min after epidural administration. If the modified Bromage score of the previous patient was >0 within 30 min after drug administration, the concentration of ropivacaine in the next patient was decreased by 1 gradient (division of the current patient’s concentration by 1.2); if the modified Bromage score of the previous patient was = 0 within 30 min after drug administration, the concentration of ropivacaine in the next patient was increased by 1 gradient (multiplication of the current patient’s concentration by 1.2). Those patients who reported ineffective analgesia at 30 min after administration of the study solution received a 5 mL bolus of 2% lidocaine administered via epidural catheter to supplement the analgesia. If the patient still reported ineffective analgesia, the patient’s data were excluded from the study analysis, and general anesthesia was used instead (21). Hypotension, defined as systolic blood pressure (SBP) < 20% of baseline value or < 90 mmHg (27), was treated with an intravenous injection of ephedrine 6 mg to restore blood pressure. Bradycardia, defined as a heart rate of less than 50 beats per minute, was treated with intravenous atropine 0.5 mg. When nausea and vomiting occurred, ondansetron 4 mg was injected intravenously.

### 2.3 Measurements

The primary outcome of this study was to ascertain the effective concentration of ropivacaine, when combined with varying doses of dexmedetomidine, for achieving motor blockade.

#### 2.3.1 Secondary outcome

General patient information was recorded. Sedation was assessed using the Ramsay sedation scale (Grade 1 = patient anxious, or agitated, or both; Grade 2 = patient cooperative, oriented, and tranquil; Grade 3 = patient responds to commands only; Grade 4 = a brisk response to a light glabellar tap; Grade 5 = a sluggish response to light glabellar tap; Grade 6 = no response) (23) at the time of the patients arriving at the operating room (T_0_), 5 min (T_1_), 15 min (T_2_), 30 min (T_3_), 1 h (T_4_), and 2 h (T_5_) after epidural administration. Systolic blood pressure (SBP), diastolic blood pressure (DBP), and heart rate (HR) were also measured and recorded at T_0_, T_1_, T_2_, T_3_, and T_4_. The level of sensory block was assessed bilaterally along the midclavicular line using the pinprick test. The onset time of sensory block was defined as the time between epidural injection and a T_10_ sensory block level being achieved, and the highest level of sensory block was also recorded. The adverse effects and complications during and 24 h after surgery, including hypotension, bradycardia, dry mouth, dizziness, nausea, and vomiting, were observed. Other indicators were recorded, such as surgery time, intraoperative fluid infusion volume and blood loss, intraoperative and postoperative use of vasoactive drugs.

### 2.4 Statistical analysis

According to published research (28), the up-down allocation method requires 20–40 study subjects to estimate the EC_50_. It is considered sufficient to obtain six pairs of reversals of sequence for a valid sample size. Based on the pre-experimental results, we calculated the sample size. When compared with the NR group, the EC_50_ values of the RD_0.25_ group showed an 8% decrease, the EC_50_ of the RD_0.5_ group a 15% decrease, the EC_50_ of the RD_0.75_ group a 33% decrease, and the EC_50_ of the RD_1.0_ group a 36% decrease. In the equation below, n symbolizes the total sample size, while P_min_ and P_max_ indicate the minimum and maximum rates, respectively. Therefore, a total sample size of 118 achieves 90% power to detect an effect size (W) of 0.3615 using a 4 degrees of freedom Chi-Square Test with a significance level (alpha) of 0.05. To account for 20% dropout rate, we thus planned to enroll 150 patients in our study. Therefore, we determined that a sample size of 30 patients for each group would be necessary to observe more than six pairs of reversals of sequence. As a backup and sensitivity analysis, probit regression analysis was conducted by analyzing the tallied numbers of “effective” and “ineffective” responses for each concentration category in each group.


$$n=\frac{1641.6\lambda }{{\left(\sin^{-1}\sqrt{P\max}-\sin^{-1}\sqrt{P\min}\right)}^{2}}$$

Statistical analysis utilized SPSS 26.0 statistical software. Continuous variables adhering to a normal distribution were presented as mean ± standard deviation ($\bar{x}$ ± s). For comparison among groups, one-way analysis of variation (ANOVA) was utilized, complemented by Bonferroni correction for *post hoc* pairwise comparison. In cases where continuous variables were measured at multiple time points within each group, two-way repeated measures ANOVA was employed, accompanied by simple effects analysis to examine any interaction effects between group and time. Variables exhibiting a skewed distribution were characterized by the median (M) and interquartile range (IQR), with the Kruskal-Wallis H test being applied for group comparisons and Bonferroni correction for ensuing pairwise comparisons. Categorical data were reported as percentiles and subjected to analysis by Pearson’s X^2^ test or Fisher’s exact test. For comparing ordinal variables across groups, the Kruskal-Wallis H test was employed, and the Friedman test was used for within-group comparisons, with Bonferroni correction facilitated for subsequent *post hoc* pairwise analyses. A *p*-value <0.05 was deemed statistically significant.

## 3 Results

**Figure 1:**
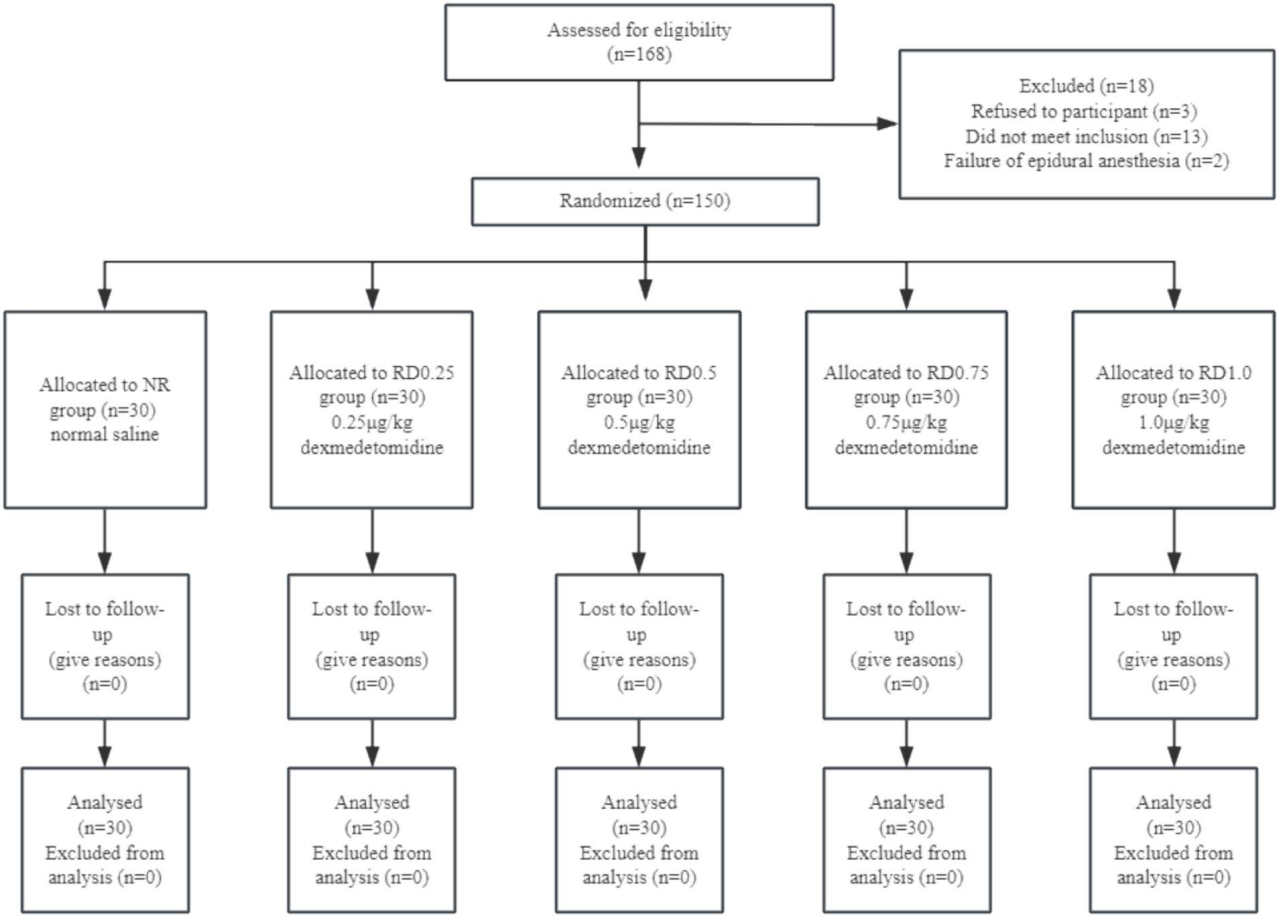
Flow diagram of study.

We recruited 168 patients for our study, 13 of them did not meet inclusion criteria, 3 of them refused participation, and 2 of them underwent a failed epidural anesthesia. Later, 150 subjects were enrolled in this study (Figure 1).

### 3.1 Demographic data and clinical characteristics

There were no significant differences in age, gender, ASA grade, weight, height, duration of surgery, fluid infusion volume, blood loss, and maximum sensory block among the five groups (*p* > 0.05). Although the overall difference in the number of people using vasoactive drugs was statistically significant (*p* < 0.05), there was no statistically significant difference in pairwise comparison among the five groups. The onset time of sensory block in the RD_0.25_, RD_0.5_, RD_0.75_, and RD_1.0_ groups was significantly shorter (*p* < 0.05), as shown in Table 1.

**Table 1:** Demographic data and clinical characteristics in five groups.

	NR group (*n* = 30)	RD_0.25_ group (*n* = 30)	RD_0.5_ group (*n* = 30)	RD_0.75_ group (*n* = 30)	RD_1.0_ group (*n* = 30)	F/χ^2^	P
Age (years)	53.0 ± 7.58	51.5 ± 8.92	53.2 ± 8.05	52.8 ± 7.71	53.1 ± 6.60	0.225	0.924
Gender (Female/Male)	27/3	28/2	26/4	27/3	26/4	1.135	0.973
ASA grade (I/II)	26/4	27/3	25/5	28/2	26/4	1.736	0.877
Weight (kg)	64.5 ± 6.7	65.2 ± 5.9	64.6 ± 8.0	66.3 ± 6.5	63.4 ± 8.1	0.646	0.630
Height (cm)	163.8 ± 5.8	165.7 ± 6.6	165.1 ± 7.4	167.1 ± 6.5	164.7 ± 6.4	1.033	0.393
Duration of surgery (min)	95.0 ± 39.7	93.2 ± 39.5	96.1 ± 36.5	93.7 ± 39.3	94.9 ± 38.3	0.027	0.999
Fluid infusion volume (mL)	838.3 ± 336.7	940.0 ± 287.2	1005.0 ± 298.1	1008.3 ± 308.5	931.7 ± 286.3	1.558	0.189
Blood loss (mL)	63.8 ± 37.8	65.3 ± 35.4	61.3 ± 35.1	64.8 ± 39.1	54.2 ± 30.3	0.497	0.738
Use of vasoactive drugs (n)	1	2	2	9	7	12.761	0.008
Maximum sensory block (T4/T6/T8)	7/22/1	6/21/3	3/24/3	2/24/4	2/23/5	8.148	0.408
Onset time of sensory block (min)	13.6 ± 2.5	11.3 ± 1.6^a^	10.6 ± 1.7^a^	11.2 ± 1.9^a^	11.2 ± 1.8^a^	12.634	0.0001

Data presented as mean ± standard deviation or number. ASA: American Society of Anesthesiologists; NR: normal saline; RD_0.25_: ropivacaine combined with 0.25 μg/kg dexmedetomidine; RD_0.5_: ropivacaine combined with 0.50 μg/kg dexmedetomidine; RD_0.75_: ropivacaine combined with 0.75 μg/kg dexmedetomidine; RD_1.0_: ropivacaine combined with 1.0 μg/kg dexmedetomidine. ^a^P < 0.05 vs. the NR group.

### 3.2 Median effective concentration

**Figure 2:**
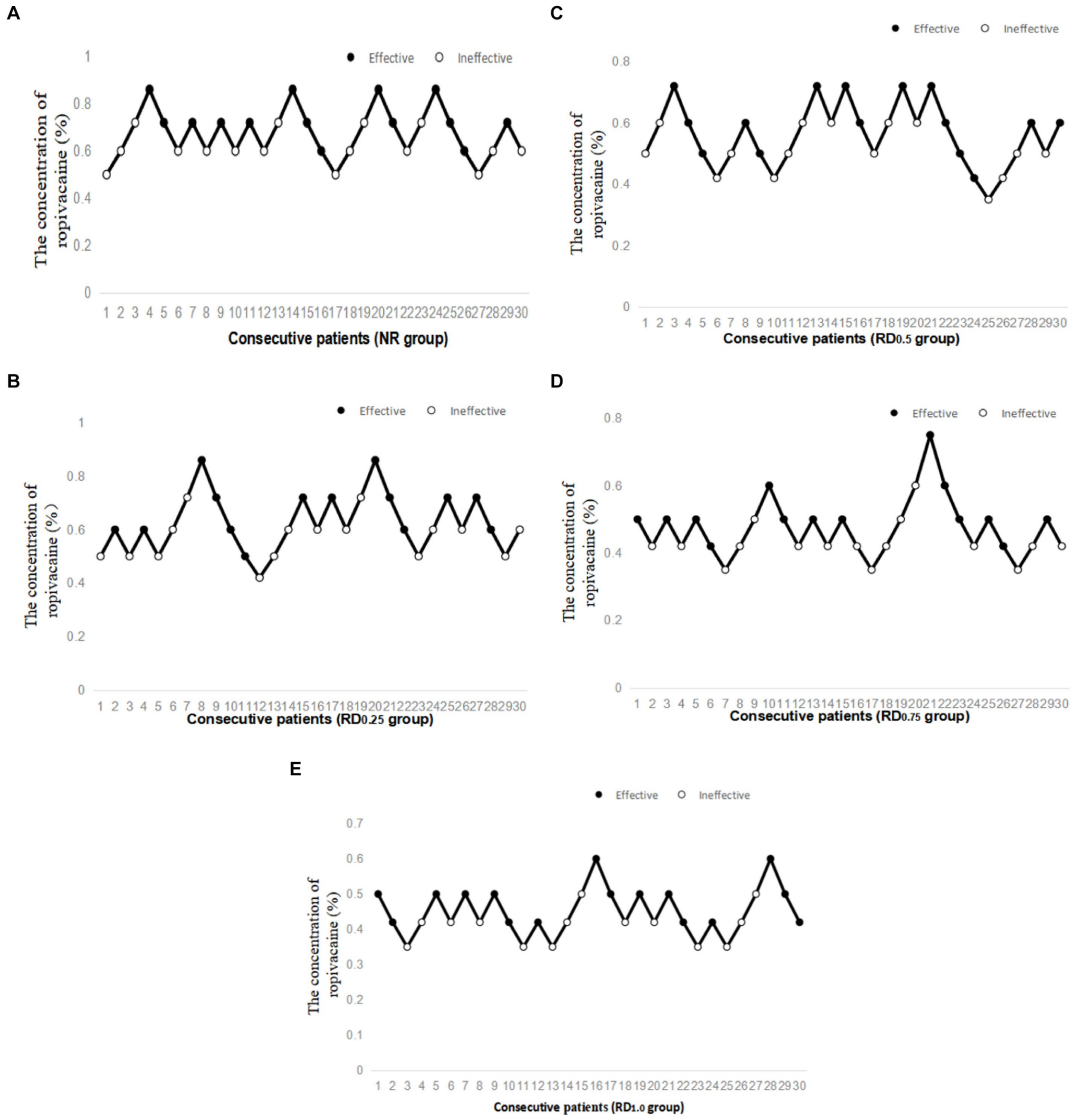
Individual response to epidural ropivacaine at corresponding concentrations (%). NR: normal saline; RD_0.25_: ropivacaine combined with 0.25 μg/kg dexmedetomidine; RD_0.5_: ropivacaine combined with 0.50 μg/kg dexmedetomidine; RD_0.75_: ropivacaine combined with 0.75 μg/kg dexmedetomidine; RD_1.0_: ropivacaine combined with 1.0 μg/kg dexmedetomidine. **(A)** Individual response to epidural ropivacaine at corresponding concentrations (%) in the NR group, **(B)** Individual response to epidural ropivacaine at corresponding concentrations (%) in the RD_0.25_ group, **(C)** Individual response to epidural ropivacaine at corresponding concentrations (%) in the RD_0.5_ group, **(D)** Individual response to epidural ropivacaine at corresponding concentrations (%) in the RD_0.75_ group and **(E)** Individual response to epidural ropivacaine at corresponding concentrations (%) in the RD_1.0_ group.

As shown in Figure 2, the EC_50_ of epidural ropivacaine required to achieve motor block was 0.677% (95% *CI*, 0.622–0.743%) in the NR group, 0.624% (95% *CI*, 0.550–0.728%) in the RD_0.25_ group, 0.549% (95% *CI*, 0.456–0.660%) in the RD_0.5_ group, 0.463% (95% *CI*, 0.408–0.527%) in the RD_0.75_ group, and 0.435% (95% *CI*, 0.390–0.447%) in the RD_1.0_ group. The EC_50_ of the NR group and the RD_0.25_ group were significantly higher than that of the RD_0.75_ and the RD_1.0_ groups (*p* < 0.05), and the EC_50_ of the RD_0.5_ group was significantly higher than that of the RD_1.0_ group (*p* < 0.05).

### 3.3 Hemodynamics

**Figure 3:**
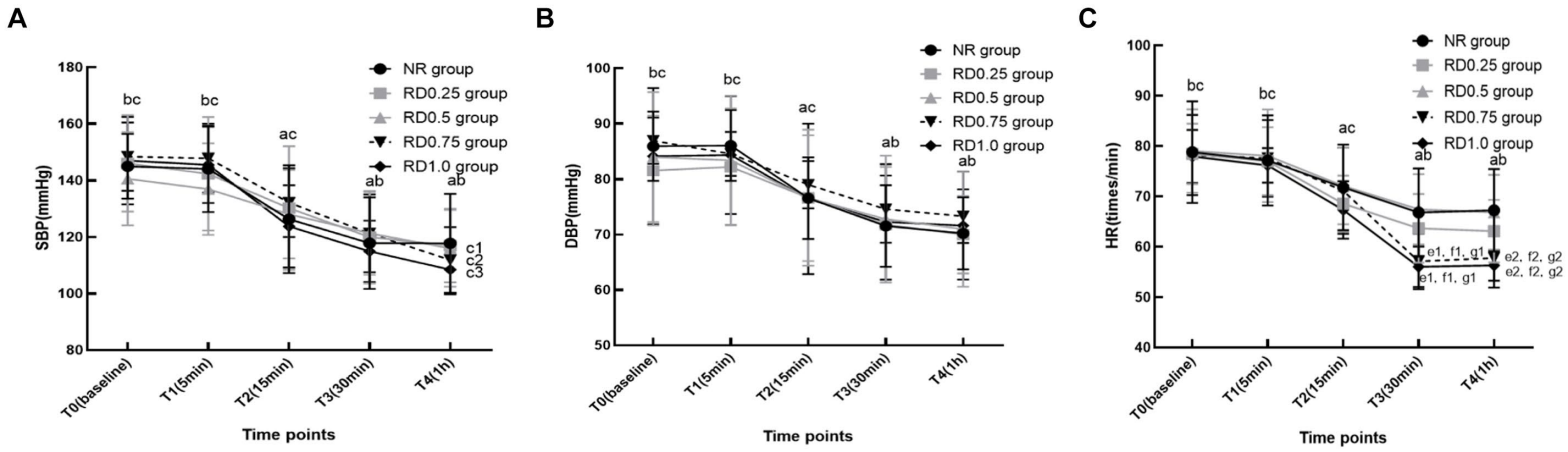
The hemodynamic changes in five groups at different time points. SBP: systolic blood pressure; DBP: diastolic blood pressure; HR: heart rate; NR: normal saline; RD_0.25_: ropivacaine combined with 0.25 μg/kg dexmedetomidine; RD_0.5_: ropivacaine combined with 0.50 μg/kg dexmedetomidine; RD_0.75_: ropivacaine combined with 0.75 μg/kg dexmedetomidine; RD_1.0_: ropivacaine combined with 1.0 μg/kg dexmedetomidine. ^a^*P* < 0.05 vs. the five groups at T_0_, *^b^P* < 0.05 vs. the five groups at T_2_, ^c^*P* < 0.05 vs. the five groups at T_3_, ^c1^*P* < 0.05 vs. the RD_0.5_ group at T_3_, ^c2^*P* < 0.05 vs. the RD_0.75_ group at T_3_, ^c3^*P* < 0.05 vs. the RD_1.0_ group at T_3_, ^d1^*P* < 0.05 vs. the NR group at T_3_, ^d2^*P* < 0.05 vs. the NR group at T_4_, ^e1^*P* < 0.05 vs. the RD_0.25_ group at T_3_, ^e2^*P* < 0.05 vs. the RD_0.25_ group at T_4_, ^f1^*P* < 0.05 vs. the RD_0.5_ group at T_3_, ^f2^*P* < 0.05 vs. the RD_0.5_ group at T_4_. **(A)** SBP, **(B)** DBP and **(C)** HR.

In the five groups, SBP, DBP, and HR significantly decreased at T_2-4_ compared to T_0_. Additionally, these parameters saw a marked reduction at T_3-4_ compared with T_2_, but exhibited a significant increase at T_0-2_ when contrasted with T_3_ (*p* < 0.05). However, in the RD_0.5_, RD_0.75_, and RD_1.0_ groups, the SBP at T_4_ was notably lower than at T_3_. Furthermore, the HR at T_3_ and T_4_ in the RD_0.75_ and RD_1.0_ groups was significantly lower compared to the NR, RD_0.25_, RD_0.5_ groups (*p* < 0.05), as depicted in Figure 3.

### 3.4 Ramsay sedation score

Compared with the NR group, Ramsay sedation score in the RD_0.25_ group was significantly higher at T_3_ (*p* < 0.05), Ramsay sedation score in the RD_0.5_ group was significantly higher at T_3-5_ (*p* < 0.05), and Ramsay sedation score in the RD_0.75_ and RD_1.0_ groups was significantly higher at T_2-5_ (*p* < 0.05). In the RD_0.25_ group, Ramsay Sedation score increased at T_3-4_ compared with T_0_, in the RD_0.5_ and RD_0.75_ groups, Ramsay score increased significantly at T_3-5_ compared with T_0_, and in the RD_1.0_ group, Ramsay score increased at T_2-5_ compared with T_0_ (*p* < 0.05), as shown in Table 2.

**Table 2:** Ramsay sedation score.

Time points	Median (interquartile range)					χ^2^	*p*
	NR group (*n* = 30)	RD_0.25_ group (*n* = 30)	RD_0.5_ group (*n* = 30)	RD_0.75_ group (*n* = 30)	RD_1.0_ group (*n* = 30)		
T0 (baseline)	2 (2–2)	2 (2–2)	2 (2–2)	2 (2–2)	2 (2–2)	3.870	0.424
T1 (5 min)	2 (2–2)	2 (2–2)	2 (2–2)	2 (2–2)	2 (2–2)	6.628	0.157
T2 (15 min)	2 (2–3)	2 (2–3)	3 (2–3)	3 (2-3)^d^	3 (3-3)^a4,d^	30.746	0.000
T3 (30 min)	2 (2–3)	3 (3–3.25)^a1,d^	3 (3-4)^a2,d^	4 (3-4)^a3,d^	4 (3-4)^a4,d^	74.424	0.000
T4 (1 h)	2 (2–3)	3 (2–3.25)^a1^	4 (3-4)^a2,d^	4 (4-4)^a3,d^	4 (4-4)^a4,d^	82.264	0.000
T5 (2 h)	2 (2–2)	2 (2–3.25)	4 (2.75-4)^a2,d^	4 (3-4)^a3,d^	4 (4-4)^a4,d^	78.138	0.000
χ^2^	27.876	84.481	110.355	131.588	133.251		
*p*	0.000	0.000	0.000	0.000	0.000		

NR: normal saline; RD_0.25_: ropivacaine combined with 0.25 μg/kg dexmedetomidine; RD_0.5_: ropivacaine combined with 0.50 μg/kg dexmedetomidine; RD_0.75_: ropivacaine combined with 0.75 μg/kg dexmedetomidine; RD_1.0_: ropivacaine combined with 1.0 μg/kg dexmedetomidine. ^a1^P < 0.05 vs. the RD_0.25_ group at T_0_, ^a2^P < 0.05 vs. the RD_0.5_ group at T_0_, ^a3^P < 0.05 vs. the RD_0.75_ group at T_0_, ^a4^P < 0.05 vs. the RD_1.0_ group at T_0_, ^d^P < 0.05 vs. the NR group.

### 3.5 Side effects

The overall differences in hypotension, bradycardia, and thirst were statistically significant (*p* < 0.05), but there was no statistically significant difference in pairwise comparison among the five groups, as shown in Table 3.

**Table 3:** Adverse effects.

	NR group (*n* = 30)	RD_0.25_ group (*n* = 30)	RD_0.5_ group (*n* = 30)	RD_0.75_ group (*n* = 30)	RD_1.0_ group (*n* = 30)	χ^2^	*P*
Hypotension	2 (6.7%)	4 (13.3%)	3 (10.0%)	10 (33.3%)	9 (30%)	11.035	0.024
Nausea and vomiting	1 (3.3%)	2 (6.7%)	1 (3.3%)	3 (10%)	4 (13.3%)	3.077	0.631
Thirst	0 (0%)	0 (0%)	2 (6.7%)	3 (10%)	5 (16.7%)	8.649	0.041
Dizziness	0 (0%)	1 (3.3%)	2 (6.7%)	1 (3.3%)	3 (10.0%)	3.544	0.602
Bradycardia	4 (13.3%)	3 (10.0%)	4 (13.3%)	11 (36.7%)	12 (40.0%)	13.396	0.08

NR: normal saline; RD_0.25_: ropivacaine combined with 0.25 μg/kg dexmedetomidine; RD_0.5_: ropivacaine combined with 0.50 μg/kg dexmedetomidine; RD_0.75_: ropivacaine combined with 0.75 μg/kg dexmedetomidine; RD_1.0_: ropivacaine combined with 1.0 μg/kg dexmedetomidine.

## 4 Discussion

This study found that dexmedetomidine as an adjuvant dose-dependently reduced the EC_50_ of epidural ropivacaine for motor block and shortened the onset time of epidural anesthesia block. 0.75 μg/kg and 1.0 μg/kg, of dexmedetomidine combined with ropivacaine resulted in a significant reduction in heart rate. However, when patients were administered with 0.5 μg/kg of dexmedetomidine combined with ropivacaine, the heart rate remained largely consistent with that of the control group. These findings suggest that the optimal dose of dexmedetomidine combined with ropivacaine for epidural anesthesia is 0.5 μg/kg.

When used as an adjuvant, dexmedetomidine combined with ropivacaine not only enhances the local anesthetic effect of ropivacaine but also reduces the EC_50_ of ropivacaine for labor analgesia (17, 22). Kazim et al. (29) found that when dexmedetomidine was added to epidural ropivacaine, patients undergoing percutaneous renal lithotripsy experienced an earlier onset of motor and sensory blockade and longer postoperative analgesia. These findings are similar to the results of our study. The EC_50_ value of ropivacaine, when combined with either 0.75 μg/kg or 1.0 μg/kg of dexmedetomidine, was significantly reduced compared to ropivacaine used alone. Furthermore, the EC_50_ value of ropivacaine with 1.0 μg/kg dexmedetomidine was distinctly lower than when combined with 0.5 μg/kg dexmedetomidine. Our findings indicated that the epidural infusion of dexmedetomidine could reduce the EC_50_ of ropivacaine for motor blockade. Furthermore, as the dosage of epidural dexmedetomidine increases, the EC_50_ for ropivacaine-induced motor blockade gradually decreases. This effect is attributed to several mechanisms: Firstly, dexmedetomidine acts as a highly selective α2 adrenoceptor agonist. Its epidural infusion affects both presynaptic and postsynaptic α2 receptors on dorsal horn neurons, leading to neuronal cell membrane hyperpolarization, which in turn produces analgesic and motor block effects (30–33). Secondly, the epidural infusion of dexmedetomidine opens K^+^ channels on the nerve cell membrane and strengthens the local anesthetics’ inhibitory action on Na^+^ channels, thereby enhancing the anesthetic effects (34). Lastly, it reduces the secretion of norepinephrine in the spinal cord, stimulates cholinergic nerves, and generates an anti-nociceptive effect (35).

Kiran et al. (30) found that compared with 0.5% ropivacaine alone, the map and heart rate of 10 μg dexmedetomidine combined with 0.5% ropivacaine decreased significantly 10 min after epidural administration. Our findings align with these results, as we observed a significant decrease in blood pressure and heart rate among the five groups of patients 15 min after epidural administration. These results indicated that dexmedetomidine could inhibit hemodynamics to a certain extent. But we also discovered that, compared with ropivacaine combined with 0.25 μg/kg and 0.5 μg/kg dexmedetomidine, the heart rate significantly decreased at 30 min and 1 h after administration when ropivacaine was used in conjunction with 0.75 μg/kg and 1.0 μg/kg of dexmedetomidine. This finding suggests that as the dosage of dexmedetomidine increases, its inhibitory effect on the heart rate intensifies, indicating a dose-dependent relationship. This is mainly because epidural infusion of dexmedetomidine can act on the α_2_ receptor of the spinal cord’s presynaptic and postsynaptic nerve endings, which causes neuronal cell membrane hyperpolarization, inhibits neuronal firing and sympathetic outflow, thereby inhibiting norepinephrine release, causing a drop in blood pressure and a slowing heart rate (36, 37).

Epidural anesthesia is a kind of awake anesthesia. The sedative effect of dexmedetomidine can not only relieve patients’ tension and anxiety, but also provide patients with good comfort (38). Kaur et al. (21) found that compared with 0.75% ropivacaine alone, the sedation score of 0.75% ropivacaine combined with 1.0 μg/kg dexmedetomidine significantly increased 30 min after epidural administration. This is similar to the results of this study, compared with ropivacaine alone, the sedation score of ropivacaine combined with 0.25 μg/kg dexmedetomidine significantly increased 30 min after epidural administration, the sedation score of ropivacaine combined with 0.5 μg/kg dexmedetomidine significantly increased at 30 min, 1 h and 2 h after epidural administration, the sedation score of ropivacaine combined with 0.75 μg/kg and 1.0 μg/kg dexmedetomidine significantly increased at 15 min, 30 min, 1 h and 2 h after epidural administration. This indicates that epidural dexmedetomidine could produce sedation. Furthermore, our study findings also reveal that with an increasing dose of epidural dexmedetomidine, the time required to achieve the same level of sedation is shorter, and the duration of the sedative effect is longer. This may be due to the fact that dexmedetomidine with high fat solubility is easily absorbed into the blood through epidural adipose tissue and acts in the brain stem α_2_ receptor, which produces sedation (39).

Soni et al. (40) found that compared with 0.75% ropivacaine alone, the anesthesia onset time of 0.75% ropivacaine combined with 1.5 μg/kg dexmedetomidine was shorter. This study also discovered that the onset time of anesthesia was shorter when dexmedetomidine was combined with ropivacaine compared to ropivacaine alone. This finding suggests that epidural dexmedetomidine has the potential to expedite the onset time of anesthesia. In our study, we found that the rates of using vasoactive drugs in the five groups were 3.3, 6.7, 6.7, 30, 23.3%, the incidences of hypotension were 6.7, 13.3, 10, 33.3, 30%, the incidences of bradycardia were 13.3, 10, 13.3, 36.7, 40%, and the incidences of thirst were 0, 0, 6.7, 10, 16.7%, respectively. The overall difference in the five groups was statistically significant (*p* < 0.05), but there was no statistically significant difference in pairwise comparison among the five groups. This may be due to our small sample size, and more randomized controlled trials are needed for further research in the future.

There are several limitations in our study. First, we can calculate the EC_50_ value by the Dixon sequential method, but the sequential method is only suitable for the calculation of half-effective measurement in studies with a small number of cases. The EC_95_ calculation, which is widely used in clinics, requires a large number of samples. Although we can calculate the EC_95_ through the EC_50_, the sequential method is the main method in this study. Therefore, it is necessary for us to explore the EC_95_ of ropivacaine for motor block in the future. Second, in this study, the onset time of anesthesia was observed, but the duration of anesthesia was not observed, which needs further study in the future. Third, we observed that the incidence of hypotension, bradycardia, and thirst significantly increased when ropivacaine was combined with 0.75 μg/kg and 1.0 μg/kg dexmedetomidine. However, the pairwise comparisons among the five groups did not reach statistical significance, likely attributable to the small sample size. To confirm these findings, a larger sample from randomized controlled trials is required.

## 5 Conclusion

Dexmedetomidine as an adjuvant for ropivacaine can dose-dependently reduce the EC_50_ of epidural ropivacaine for motor block and shorten the onset time of epidural ropivacaine block. 0.75 μg/kg and 1.0 μg/kg of dexmedetomidine combined with ropivacaine resulted in a significant reduction in heart rate. However, when patients were administered with 0.5 μg/kg of dexmedetomidine combined with ropivacaine, the heart rate remained largely consistent with that of the control group. Therefore, the optimal dose of dexmedetomidine combined with ropivacaine for epidural anesthesia was 0.5 μg/kg.

## Data availability statement

The datasets presented in this study can be found in online repositories. The names of the repository/repositories and accession number(s) can be found at: http://www.chictr.org.cn/listbycreater.aspx.

## Ethics statement

The studies involving humans were approved by the Ethics Committee of the Affiliated Hospital of North Sichuan Medical College. The studies were conducted in accordance with the local legislation and institutional requirements. The participants provided their written informed consent to participate in this study.

## Author contributions

J-XW: Conceptualization, Data curation, Formal analysis, Funding acquisition, Investigation, Methodology, Project administration, Resources, Validation, Visualization, Writing – original draft, Writing – review & editing, Software, Supervision. CL: Conceptualization, Data curation, Formal analysis, Funding acquisition, Methodology, Validation, Visualization, Writing – original draft. Z-QW: Conceptualization, Data curation, Investigation, Methodology, Project administration, Resources, Visualization, Writing – original draft. DF: Conceptualization, Data curation, Formal analysis, Investigation, Methodology, Project administration, Resources, Writing – original draft. YW: Conceptualization, Formal analysis, Funding acquisition, Investigation, Methodology, Project administration, Resources, Writing – original draft. F-JW: Conceptualization, Data curation, Formal analysis, Funding acquisition, Investigation, Methodology, Project administration, Resources, Software, Supervision, Validation, Visualization, Writing – original draft, Writing – review & editing.
